# Association of muscle strength and body mass index with risk factors for metabolic syndrome and its prevalence in Korean adult women

**DOI:** 10.1186/s12889-022-14520-y

**Published:** 2022-11-10

**Authors:** Ju-hak Lee, Soon-young Kim, Dong-il Kim

**Affiliations:** 1 Department of Human Movement Science, Incheon National University, Incheon, Republic of Korea; 2 Department of Physical Education, College of Arts and Physical Education, Gachon University, Seongnam, Republic of Korea; 3 Sports Functional Disability Institute, Incheon National University, Incheon, Republic of Korea; 4 Division of Health and Kinesiology, Incheon National University, Incheon, Republic of Korea; 5 Sport Science Institute, Incheon National University, Incheon, Republic of Korea

**Keywords:** Metabolic syndrome (MetS), Handgrip strength, BMI (body mass index), Adult women population, Physical activity, Exercise

## Abstract

**Background:**

The aim of this study was to investigate the effects of muscle strength and BMI (body Mass Index) on Metabolic syndrome (MetS) risk factors and prevalence in Korean adult women, using data from the Korea National Health and Nutrition Examination Survey.

**Methods:**

A total of 3189 Korean adults women participated in the cross-sectional study. Participants were measured BMI, MetS risk factors including waist-circumference (WC), fasting glucose (FG), triglyceride (TG), high density lipoprotein cholesterol (HDL-C), and handgrip strength as muscle strength.

**Results:**

As a result ‘high BMI & Low muscle strength’, ‘low BMI & low muscle strength’, and ‘high BMI & high muscle strength’ groups had a significantly higher prevalence of Mets [OR (Odd ratio): 1.49, 95% CI (confidence interval): 1.01 2.20; OR: 5.77, 95% CI: 4.32 7.17; OR: 10.46, 95% CI: 8.05 13.59] than ‘low BMI & high muscle strength’ group; and after adjusting smoking, menstruation status, and drinking rate, the OR were 1.07 (95% CI: 0.71–1.61), 4.89 (95% CI: 3.60–6.55), and 7.38 (95% CI: 5.63–9.68), respectively.

**Conclusions:**

These findings indicated that increasing muscle strength and lowering BMI through regular physical activity and exercise are effective methods to reduce the prevalence of risk factors for Mets.

## Introduction

The World Health Organization (WHO) defines obesity or overweight as abnormal or excessive fat accumulation that presents a risk to health [1]. The prevalence of obesity is continuously increasing because of increased sedentary lifestyles and unhealthy lifestyles and eating habits in westernized societies as well as developing countries [2, 3]. In 2015, 39% of the world’s population was reportedly obese or overweight. In particular, the prevalence of obesity has been reported to be higher in women aged ≥19 years than in men aged ≥19 years [4]. Taken together, obesity is a major public health issue in the twenty-first century and is emerging as an unresolved problem [1].

Obesity is a major cause of diabetes, and it increases insulin resistance [5] and the incidence of various chronic diseases [6, 7]. In particular, obesity considerably influences the risk factors for metabolic syndrome (Mets) [8]. In addition, Mets is related to women’s menstruation, smoking, and drinking [9–11]. Mets increases the risk of various diseases such as diabetes, stroke, and myocardial infarction, and it greatly affects the quality of life and causes health problems [12, 13]. Regular physical activity or exercise lowers the risk of development of various chronic diseases such as obesity and Mets, and it has protective effects against risk factors affecting the prevalence of various chronic diseases [14, 15]. Delgado-Floody et al., reported that a 20-week resistance exercise training program significantly reduced waist circumference (WC), systolic blood pressure (SBP), and triglycerides (TG) in 21 subjects with obesity [body mass index (BMI) ≥ 35.0–39.9 kg/m^2^] or severe obesity (BMI ≥ 40 kg/m^2^). In particular, significant decreases in diastolic blood pressure (DBP) and increases in high-density lipoprotein-cholesterol (HDL-C) were observed in subjects with severe obesity [16].

Resistance exercise was reported to have a positive effect on Mets and various diseases [17]. Resistance exercise training is also effective in increasing muscle mass [18] and, consequently, muscle strength [19]. There are various studies regarding the association between Mets and muscle strength [20, 21]. de Lima et al., reported that Mets and muscle strength were directly related, and that the higher the strength level, the lower the risk of Mets [22]. Atlantis et al. reported that the prevalence of Mets was 2.15 times higher in the low grip force group than in the high grip force group [23]. Also, Lopez-Lopez et al. reported that the risk of metabolic syndrome was 1.39 times higher in the low grip strength group than in the high grip strength group [24]. Tomeleri et al., evaluated the risk factors for Mets in 53 older individuals and found that, compared with the values in the group without resistance training, fasting glucose (FG), WC, and SBP were significantly reduced in the group that participated in a 12-week resistance training program. Moreover, in the resistance training group, FG and SBP after the 12-week exercise program significantly reduced relative to those before the program [25]. As such, it has been reported that increased muscle strength through resistance exercise training has protective effects against risk factors for Mets. Therefore, resistance exercise training is essential to lower the risk factors and prevalence of Mets.

Various published studies regarding Mets associated with obesity in adult women have been conducted in foreign countries [26, 27]. However, the number of related studies in Korea is limited; in particular, large-scale cohort studies involving Korean adult women are scarce. Therefore, the aim of this study was to investigate the effects of BMI and muscle strength assessed by handgrip strength, on risk factors for Mets in Korean adult women, using data from the Korea National Health and Nutrition Examination Survey.

## Materials and methods

### Subjects

This study was conducted using the raw data from the 2019 Korea National Health and Nutrition Examination Survey published in 2021. From 8110 participants in the 2019 Korea National Health and Nutrition Examination Survey, 3189 adult women aged ≥19 years who underwent assessments of handgrip strength, BMI, and risk factors for metabolic syndrome WC, SBP, DBP, FG, TG, HDL-C were selected and analyzed. The characteristics of the included subjects are shown in Table 1.

**Table 1:** Characteristics of Participants

Variables	High Muscle strength (*n* = 1585)	Low Muscle strength (*n* = 1604)	*p*-value
Age (years)	48.50 ± 15.10	55.83 ± 17.04	< 0.001
Height (cm)	158.75 ± 6.22	156.94 ± 6.71	< 0.001
Weight (kg)	55.28 ± 7.50	61.63 ± 10.71	< 0.001
BMI (kg/m^2^)	21.94 ± 2.77	25.00 ± 3.87	< 0.001
WC (cm)	76.51 ± 8.19	85.28 ± 9.99	< 0.001
SBP (mmHg)	115.96 ± 16.77	121.08 ± 18.12	< 0.001
DBP (mmHg)	74.06 ± 9.08	74.77 ± 9.47	0.032
TG (mg/dl)	102.52 ± 66.93	124.50 ± 77.49	< 0.001
TC (mg/dl)	195.41 ± 36.98	194.50 ± 38.87	0.496
Glucose (mg/dl)	96.53 ± 19.45	102.17 ± 23.06	< 0.001
HDL-C (mg/dl)	58.30 ± 12.68	53.66 ± 12.49	< 0.001
LDL-C (mg/dl)	116.60 ± 33.71	115.94 ± 35.35	0.587
Relative Grip (kg/kg)	44.91 ± 5.33	30.54 ± 5.51	< 0.001

Values are Mean ± SD, *WC* waist circumference, *BMI* Body mass index, *SBP* Systolic blood pressure, *DBP* Diastolic blood pressure, *TG* Triglycerides, *TC* Total cholesterol, *HDL-C* High density lipoprotein cholesterol, *LDL-C* Low density lipoprotein cholesterol

### Handgrip strength and anthropometric measurements

In this study, handgrip strength was evaluated according to the recommendations of the Institute of Medicine.

[28]. Handgrip strength was measured using a digital grip strength dynamometer (T.K.K. 5401, TAKEI, Niigata, Japan) to measure determine muscle strength. Handgrip strength was measured by asking each subject to hold a hand-held dynamometer for 3 seconds while keeping the feet wide apart in line with the width of the pelvis and looking straight ahead. The back and shoulders were kept straight, and a straight posture was maintained without the arms touching the body. Then, the relative handgrip strength (handgrip strength/weight × 100) for the dominant hand of each subject was measured to determine the subject’s muscle strength. Each subject’s height and weight were measured using a stadiometer (seca 274, seca, Hamburg, Germany) and a weighing scale (GL-6000-20, G-tech, Seoul, Korea), respectively [29].

### Assessment of risk factors for metabolic syndrome

Assessment of risk factors for metabolic syndrome measure were performed by a specially trained examiner who followed a standard procedure. The risk factors for Mets in this study were measured as follows. WC was measured to one decimal place (0.1 cm) using a measuring tape (Lufkin W606PM, Lufkin, Michigan, USA) at the narrowest point between the lowest rib and the upper iliac crest after exhalation. Blood pressure was measured 3 times on the right arm in total using a mercury sphygmomanometer (Baumanometer, W.A.Baum, New York, USA) in a sitting position after 5 minutes of rest. Blood test samples were directly collected by a qualified nurse after an 8-hour fasting period, and blood analysis for risk factors for Mets was performed according to the Korea National Health and Nutrition Examination Survey guidelines.

### Definition of the criteria for metabolic syndrome

The criteria for metabolic syndrome were defined on the basis of the diagnostic criteria suggested in the NCEP-ATPIII in 2001 [30]. According to the NCEP-ATPIII, an individual is diagnosed with metabolic syndrome if he or she has at least three of the five risk factors: FG ≥ 110 mg/dL, TG ≥ 150 mg/dL, HDL-C < 40 mg/dL in men or < 50 mg/dL in women, DBP ≥ 85 mmHg or SBP ≥ 130 mmHg, and WC ≥ 90 cm in Asian men or ≥ 80 cm in Asian women [31].

### Covariates

The covariates in this study were age, smoking, menstrual status, and drinking rate. Age was obtained through a health survey questionnaire. Smoking, menstrual status and drinking rate were obtained through health behavior survey questionnaire. Current smoking was investigated for smoking, and Menstruation status was investigated whether the current menstruation. The drinking rate was investigated by dividing the number of subjects who participated in the questionnaire by those who drinking at least once a month for the past 1 year.

### Data analysis

All data were statistically analyzed using SPSS/Window 25.0. To investigate the effects of BMI and muscle strength on the risk factors for Mets and its prevalence, the subjects were divided into a normal weight group (BMI < 23 kg/m^2^) and an overweight group (BMI ≥ 23 kg/m^2^) according to the BMI criteria published by the WHO in 2000 [32]. Subjects were divided into groups based on the median value of the relative handgrip strength [33–35]. The muscle strength according to the relative grip strength level was divided into the high muscle strength level of the top 50% and the low muscle strength level of the bottom 50% based on the median (37.8 kg) of the relative grip strength of all subjects. Between-group differences in the mean value of each measurement item were analyzed using an independent t-test. In addition, in order to investigate the relationship between the Mets risk factors according to the muscle strength level and BMI (23 kg/m ^2^), subjects were divided into high and low groups according to the muscle strength after adjusting for age, and BMI after adjusting for age and menstruation status. Then, between-group differences in the measured risk factors were analyzed using analysis of covariance (ANCOVA). The subjects were divided into 4 groups according to BMI level and the muscle strength which is divided by the relative handgrip strength level: 1) low BMI & high muscle strength group, 2) low BMI & low muscle strength group, 3) high BMI & high muscle strength group, 4) They were divided into high BMI & low strength groups. Four groups compared and analyzed the differences in the effects of Mets risk factors between groups using ANCOVA. Finally, in order to compare and analyze the risk of Mets, age, smoking status, drinking rate, and menstruation status which affect the prevalence of Mets, were controlled, and then compared and analyzed using logistic regression. The significance level was set at *p* < 0.05.

## Results

### The effect of BMI and muscle strength on risk factors of metabolic syndrome

The subjects of this study were divided into high and low groups according to muscle strength (assessed using handgrip strength) and BMI, and the risk factors for Mets were analyzed and compared as shown in Table 2. After adjusting for age, smoking, menstruation status and drinking rates, WC (*p* < 0.001), TG (*p* < 0.001), and FG (*p* < 0.001) were found to be significantly lower in the high muscle strength group than in the low muscle strength group, while HDL-C (*p* < 0.001) was significantly higher in the high muscle strength group. After adjusting for age, smoking, menstruation status and drinking rates, WC (*p* < 0.001), SBP (*p* < 0.001), DBP (*p* < 0.001), TG (*p* < 0.001), and FG (*p* < 0.001) were significantly lower, while HDL-C (*p* < 0.001) was significantly higher, in the low BMI group than in the high BMI group.

**Table 2:** The Effect of BMI and Muscle strength on Risk factors of metabolic syndrome

Total (*n* = 3189)	*Muscle strength levels			#BMI levels		
	High Muscle strength (*n* = 1604)	Low Muscle strength (*n* = 1,.585)	*p*-value	High BMI (*n* = 1800)	Low BMI (*n* = 1389)	*p*-value
WC (cm)	77.23 ± 0.22	84.56 ± 0.22	< 0.001	86.57 ± 0.16	73.49 ± 0.19	< 0.001
SBP (mmHg)	118.05 ± 0.37	118.96 ± 0.38	0.091	119.88 ± 0.35	116.73 ± 0.40	< 0.001
DBP (mmHg)	74.19 ± 0.23	74.64 ± 0.23	0.178	75.29 ± 0.22	73.27 ± 0.22	< 0.001
TG (mg/dl)	105.23 ± 1.80	121.77 ± 1.81	< 0.001	124.37 ± 1.66	94.11 ± 1.90	< 0.001
HDL-C (mg/dl)	57.64 ± 0.31	54.33 ± 0.31	< 0.001	53.55 ± 0.29	59.16 ± 0.33	< 0.001
Glucose (mg/dl)	97.78 ± 0.52	100.90 ± 0.52	< 0.001	101.95 ± 0.49	95.95 ± 0.56	< 0.001

Values are Mean ± SE, *WC* Waist circumference, *SBP* Systolic blood pressure, *DBP* Diastolic blood pressure, *TG* Triglycerides, *HDL-C* High density lipoprotein cholesterol, adjusted for age, smoking, menstruation status and drinking rates

### Risk factors for metabolic syndrome and its prevalence according to BMI and muscle strength

**Fig. 1:**
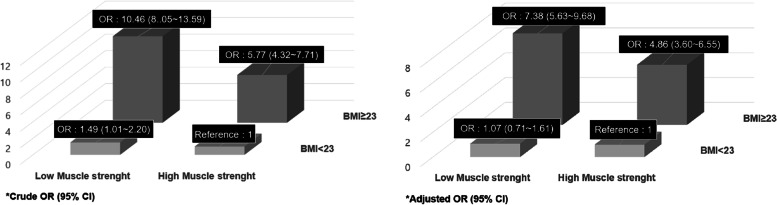
Odd Ratio according to Muscle Strength and BMI

The subjects were divided into four groups for comparison. After adjusting for age, the risk factors for Mets according to BMI and muscle strength were analyzed (Table 3). The results showed that WC (*p* < 0.001), SBP (*p* < 0.001), DBP (*p* < 0.001), TG (*p* < 0.001), FG (*p* < 0.001), and the prevalence of Mets (*p* < 0.001) were significantly lower in the low BMI & high muscle strength group than in the high BMI & low muscle strength group, whereas HDL-C (*p* < 0.001) was significantly higher in the low BMI & high muscle strength group than in the high BMI & low muscle strength group. In addition, analysis of the prevalence of Mets (Table 4) showed that the prevalence was significantly higher by 1.49 [OR (Odd ratio): 1.49, 95% CI (confidence interval): 1.01–2.20], 5.77 (OR: 5.77, 95% CI: 4.32–7.71), and 10.46 (OR: 10.46, 95% CI: 8.05–13.59) times in the low BMI & low muscle strength, high BMI & high muscle strength, and high BMI & low muscle strength groups, respectively, than in the high muscle mass & low BMI group. After adjusting for age, smoking, menstruation status, and drinking rate, the prevalence of Mets was significantly higher by 4.86 (OR: 4.86, 95% CI: 3.60–6.55) and 7.38 (OR: 7.38, 95% CI: 5.63–9.68) times in the high BMI & high muscle strength and high BMI & low muscle strength groups, respectively, than in the low BMI & high muscle strength group (Fig. 1).

**Table 3:** Risk Factors and Prevalence of Metabolic Syndrome according to BMI and Muscle Strength

Total (*n* = 3189)	BMI < 23 kg/m^2^		BMI ≥ 23 kg/m^2^	
	High Muscle strength (*n* = 986)	Low Muscle strength (*n* = 403)	High Muscle strength (*n* = 618)	Low Muscle strength (*n* = 1182)
WC (cm)	72.88 ± 0.22	74.76 ± 0.33*	83.57 ± 0.27*#	88.22 ± 0.20*#†
SBP (mmHg)	116.95 ± 0.48	116.19 ± 0.73	119.66 ± 0.59*#	119.99 ± 0.44*#
DBP (mmHg)	73.35 ± 0.30	73.09 ± 0.46	75.41 ± 0.37*#	75.22 ± 0.27*#
TG (mg/dl)	91.63 ± 2.27	99.61 ± 3.46	125.23 ± 2.80*#	130.21 ± 2.06*#
HDL-C (mg/dl)	59.87 ± 0.39	57.52 ± 0.60*	54.32 ± 0.48*#	53.11 ± 0.36*#
Glucose (mg/dl)	96.02 ± 0.67	95.68 ± 1.02	100.32 ± 0.82*#	102.83 ± 0.61*#
**Metabolic Syndrome**				
No. of participants with Metabolic syndrome (%)	75/986 (7.61)	44/403 (10.92)	199/618 (32.20)*#	547/1182 (46.28)*#†

Values are mean ± SE, *WC* Waist circumference, *SBP* Systolic blood pressure, *DBP* Diastolic blood pressure, *TG* Triglycerides, *HDL-C* High density lipoprotein cholesterol, *significantly different from first group, #significantly different from second group, †significantly different from third group, adjusted for age, smoking, menstruation status and drinking rates. *p* < 0.05

**Table 4:** Risk Rate of Metabolic Syndrome According to BMI and Muscle Strength

Total (*n* = 3189)	BMI < 23 kg/m^2^		BMI ≥ 23 kg/m^2^	
	High Muscle strength (*n* = 986)	Low Muscle strength (*n* = 403)	High Muscle strength (*n* = 618)	Low Muscle strength (*n* = 1182)
**Metabolic syndrome**				
OR (95% CI:)	1	1.49(1.01 ~ 2.20)	5.77(4.32 ~ 7.71)	10.46(8.05 ~ 13.59)
Adjusted OR (95% CI:)	1	1.07(0.71 ~ 1.61)	4.86(3.60 ~ 6.55)	7.38(5.63 ~ 9.68)

Abbreviations: *OR* Odds Ratio, *CI* confidence interval, adjusted for age, smoking, menstruation status and drinking rates

## Discussion

In this study, we investigated the effects of BMI and muscle strength on risk factors for Mets and its prevalence in Korean adult women aged 19 years or older using data from the 2019 Korea National Health and Nutrition Examination Survey. The results showed that WC, TG and FG were significantly lower, while HDL-C was significantly higher, in the high muscle strength group than in the low muscle strength group. The muscle strength was evaluated by measuring handgrip strength in this study. It has been reported that muscle strength assessed by handgrip strength is closely associated with the prevalence of and mortality from various diseases [36]. In particular, it has been reported that the level of muscle strength is closely associated with risk factors for Mets [21], and that lower muscle strength increases the risk factors for Mets and mortality associated with Mets [20, 37]. In addition, Agner et al., reported that high muscle strength is an important factor in lowering the risk factors and prevalence of Mets [38].

Ji et al., evaluated 2521 women and analyzed the association between risk factors for Mets and muscle strength assessed by the relative handgrip strength according to body weight, after adjusting for age, race, drinking, smoking, education level, income, energy intake, and physical activity. As a result, when muscle strength was low, WC, TG, FG, SBP, and DBP were high. Also, HDL-C was low [39]. In this regard, it is known that muscle strength is closely associated with muscle mass [40], and an increase in muscle mass can be improved through resistance exercise training; this indicates that increased muscle strength protects against the risk factors for Mets [19]. Oliveira et al., reported that a 1-week resistance exercise training program had protective effects against total cholesterol (TC), TG, and WC in 22 menopausal women [41].

When subjects were stratified by BMI, we found that WC, SBP, DBP, TG, and FG were significantly lower, while HDL-C was significantly higher, in the low BMI group than in the high BMI group. High BMI is negatively affects health and is known to be a major risk factor for various diseases, including Mets [42, 43]. Balgoon et al., evaluated the association between BMI and risk factors for Mets in 165 women and reported that risk factors such as WC, SBP, and DBP were significantly higher in women with a high BMI (≥25 kg/m^2^) than in those with a low BMI (< 25 kg/m^2^), and that the risk of Mets increased with BMI [44]. In addition, Choromańska et al., evaluated 44 women and reported that WC, BP, FG, and TG were significantly lower, while HDL-C was significantly higher, in non-obese women than in obese women with Mets. These results indicate that a high BMI negatively affects Mets and increases its prevalence [45]. Therefore, adequate control of BMI can have protective effects against risk factors for Mets.

Finally, in this study, we found that WC, SBP, DBP, TG, and FG were significantly lower, while HDL-C was significantly higher, in subjects with low BMI and high muscle strength than in those with high BMI and low muscle strength. Moreover, the prevalence of Mets was 10.46 times higher in the high BMI & low muscle strength group than in the low BMI & high muscle strength group; after adjusting for age, menstruation status, smoking and drinking rates, the prevalence was 7.38 times higher in the high BMI & low muscle strength group. Similarly, Lu et al., analyzed the risk of Mets development according to muscle strength and obesity after adjusting for age, sex, smoking, and drinking and reported that subjects with low BMI and obesity had an 11.93 times higher risk than did subjects with normal weight [46]. In addition, Takayama et al., reported that the prevalence of Mets was 3.12 times higher in subjects with low muscle strength and high BMI than in those with normal muscle strength and weight after adjusting for age and sex [47]. Song et al., evaluated the association between the prevalence of Mets and handgrip strength and investigated the prevalence of Mets according to handgrip strength and BMI in 542 elderly women. They found that with a decrease in handgrip strength and increase in BMI, there was a 2.25-fold increase in the prevalence of Mets. This study also showed that lower handgrip strength and a higher BMI were associated with a 2.12-fold increase in the prevalence of Mets after adjusting for age, smoking status, occupation, education level, family income, nutritional status, and physical activity [21]. As such, it can be said that muscle strength and BMI have a strongly associated with Mets risk factors and prevalence. Therefore, it is thought that the prevalence of Mets can be lowered by lowering BMI through regular exercise and physical activity and increasing muscle strength through resistance exercise training.

This study has some limitations. First, because the subjects were only Korean adult women, the results cannot be generalized to adult women of different races and adult men. Second, because the subjects were aged ≥19 years, the results cannot be applied to adolescents and children aged < 19 years old. Third, because BMI levels was Asian standards (BMI ≥ 23 kg/m^2^), the results cannot be extended to countries other than Asian countries. Fourth, BMI is calculated by dividing weight by the square meter of height (BMI = kg/m^2^) and is used as an indicator of obesity, but it is difficult to obtain accurate body composition including muscle. Fifth, since this study did not consider the participation rate of resistance exercise to improve muscle strength related to muscle mass, additional research is needed on the prevalence of metabolic syndrome according to resistance exercise participation and strength level and BMI level. Sixth, WC and Mets are closely related [48, 49], but in this study, the effect of WC was not analyzed because it was a study to investigate the effect of muscle strength level and BMI level on metabolic syndrome. Therefore, studies related to WC will be analyzed later. Finally, because this study determined the association of BMI and muscle strength based on handgrip strength with risk factors for Mets in Korean adult women, it is difficult to broadly apply the results to determine a causal association. Despite these limitations, this study is significant in that it comparatively analyzed a large number of samples using data from the first year of the 8th Korea National Health and Nutrition Examination Survey in 2019 and confirmed the effects of BMI and muscle strength based on handgrip strength on risk factors for Mets and its prevalence.

## Conclusions

This study confirmed BMI and muscle strength in Korean adult women had an effect on Mets risk factors and prevalence. Therefore, increasing muscle strength and lowering BMI through regular resistance exercise and physical activity is a very effective way to reduce the risk factors and prevalence of Mets.

## Data Availability

This study utilized data from the Korea National Health and Nutrition Examination Survey publicly available from the Korea Disease Control and Prevention Agency. This data can be accessed through a link: https://knhanes.kdca.go.kr/knhanes/main.do

## Abbreviations

BMI: Body mass index
Mets: Metabolic syndrome
OR: Odd ratio
CI: Confidence interval
WC: Waist-circumference
FG: Fasting glucose
TG: Glucose triglyceride
HDL-C: High density lipoprotein cholesterol
SBP: Systolic blood pressure
DBP: Diastolic blood pressure
WHO: The World Health Organization
ANCOVA: Analysis of covariance
TC: Total cholesterol
